# DEPENDENCY IN ACTIVITIES OF DAILY LIVING: THE ROLE OF MULTIDIMENSIONAL FRAILTY AND PROTECTIVE FACTORS

**DOI:** 10.1093/geroni/igz038.2367

**Published:** 2019-11-08

**Authors:** G A Rixt Zijlstra, Anne van der Vorst, Linda P M Op het Veld, Nico De Witte, Jos M G A Schols, Gertrudis Kempen

**Affiliations:** 1 Maastricht University, Care and Public Health Research Institute (CAPHRI), Department of Health Services Research, Maastricht, Netherlands; 2 Zuyd University of Applied Sciences, Faculty of Health, Centre of Research Autonomy and Participation for Persons with a Chronic Illness, Heerlen, Netherlands; 3 Vrije Universiteit Brussel, Brussel, Belgium

## Abstract

Most older adults prefer to “age in place” and maintain independent regarding activities of daily living (ADL). Dependency in ADL might be caused by frailty. This study explored the relationship between multidimensional frailty and ADL dependency, and if protective factors, derived from a systematic literature review, moderate this relationship. A longitudinal study with a 24-month follow-up was performed among 1,027 community-dwelling older adults. Multidimensional frailty was assessed with the Tilburg Frailty Indicator, and ADL dependency with the Groningen Activity Restriction Scale. Other measures included socio-demographic characteristics and seven protective factors against ADL dependency, such as physical activity and non-smoking. Logistic regression analyses showed that frail older people had a twofold risk of developing ADL dependency in comparison to non-frail older people after 24 months (OR = 2.12, 95% CI = 1.50-3.00). Analyses with interaction terms indicated that the selected protective factors against ADL dependency did not signiﬁcantly moderate this relationship. Nonetheless, higher levels of physical activity and having suﬃcient ﬁnancial resources decreased the risk of becoming ADL dependent in the overall sample (OR = 0.67, 95% CI = 0.46-0.98 and OR = 0.49, 95% CI = 0.35-0.71, respectively). In conclusion, multidimensional frail older people are at higher risk of developing ADL dependency and the studied factors against ADL dependency did not signiﬁcantly moderate this relationship. To develop prevention strategies for ADL dependency and facilitate aging in place, future studies might explore the relationship between each specific frailty domain and ADL dependency, and the role of (other) moderating factors.

